# Deep Analysis of Imaging Characteristics of Spaceborne SAR Systems as Affected by Antennas Using 3D Antenna Pattern

**DOI:** 10.3390/s25195969

**Published:** 2025-09-25

**Authors:** Wei Shi, Heqing Huang, Wenjun Gao, Huaian Zhou, Hua Jiang

**Affiliations:** 1 Beijing Institute of Spacecraft System Engineering, Beijing 100094, China; littlestone_buaa@163.com (W.S.); 13521874144@139.com (W.G.); bricker99@sina.com (H.Z.); 2 School of Electronic and Information Engineering, Beihang University, Beijing 100191, China; huangheqing@buaa.edu.cn

**Keywords:** synthetic aperture radar, antenna system, SAR imaging, high resolution

## Abstract

Spaceborne Synthetic Aperture Radar (SAR) has become an indispensable tool for environmental monitoring, offering all-weather, day-and-night imaging capabilities. Before the launch, accurately analyzing the imaging characteristics of spaceborne SAR systems on the ground is crucial, and the antenna system is a very important part of SAR system simulation. This paper investigates the impact of antenna configuration on SAR imaging characteristics by using 3D antenna pattern, focusing on resolution consistency, coverage uniformity, and system adaptability under varying observation geometries. Different from the traditional SAR simulation with 2D antenna pattern (range direction and azimuth direction antenna pattern), we provide a novel simulation method by using 3D antenna pattern, which increases the simulation accuracy and realism. The two mainstream spaceborne SAR antennas (phased array antenna (PAA) and reflector antenna (RA)) are used to illustrate the differences between 2D antenna pattern and 3D antenna pattern. We provide a comparative analysis in the context of high-resolution and wide-swath imaging missions. Additionally, the importance of integrating 3D antenna pattern into SAR system simulation is emphasized, as it improves simulation fidelity, reduces development risk, and supports design validation. This study provides insights for the design and optimization of future SAR system simulation.

## 1. Introduction

Synthetic Aperture Radar (SAR) satellites [1,2,3] employ active microwave sensing techniques, involving both signal transmission and reception. By applying range pulse compression in the range direction and synthetic aperture techniques in the azimuth direction, SAR systems are capable of penetrating clouds, rain, fog, sandstorms, and other obscurants, thereby enabling all-weather, day-and-night imaging capabilities. SAR satellites enable high-resolution, wide-swath imaging, interferometric measurements for height estimation, and monitoring of subtle surface deformations, making them the most effective means of data acquisition in perennially cloudy and rainy regions. Unlike optical remote sensing, SAR satellites can acquire complex images of the observed area, simultaneously containing intensity and phase information. Through Interferometric Synthetic Aperture Radar (InSAR) techniques [4,5], phase information from radar complex image data can be extracted to infer terrain and surface micro-change information. These capabilities endow SAR satellites with unique application value across a wide range of fields, including natural resource management, geology, seismic monitoring, disaster prevention and mitigation, agriculture, forestry, hydrology, cartography, and military operations. As a result, SAR technology has garnered significant attention from governments and research institutions worldwide, driving rapid advancements in its development [6,7,8,9,10,11,12].

Since the beginning of the 21st century, the rapid development and widespread application of SAR technology have led to an increasingly urgent demand for high-resolution [13] and wide-swath [14] imaging. This demand is driven by the continuous pursuit of more detailed information about the Earth’s surface. As a result, research into methods for achieving high-resolution, wide-swath SAR imaging has become a critical focus. Within this context, the antenna system [15,16]—a core component of spaceborne SAR platforms—plays a pivotal role in imaging performance. It is responsible for precisely directing high-power microwave energy toward the target area, and its performance directly determines the quality and effectiveness of SAR imaging. In particular, meeting the dual requirements of high resolution and wide swath imposes significant challenges on antenna system design and implementation [17,18,19,20]. Traditional antenna technologies encounter several limitations in fulfilling the dual requirements of high-resolution and wide-swath imaging. For example, single-channel systems often fail to achieve high azimuth resolution, and there exists an inherent trade-off between antenna size and beamwidth. To address these challenges, advanced antenna configurations have been increasingly adopted in modern SAR systems. These novel designs typically incorporate multiple transmit/receive channels, enabling the simultaneous realization of high azimuth resolution and wide swath coverage in the range direction, thereby significantly enhancing the overall imaging capability and performance of SAR systems.

Two primary types of spaceborne SAR antennas are phased array antenna (PAA) [21,22] and reflector antenna (RA) [23,24,25,26,27], each implemented using different configurations: (1) distributed transmit/receive (T/R) modules for PAA, and (2) centralized high-power transmitters for RA systems. High-resolution, wide-swath imaging requires PAAs to support flexible and precise 2D electronic beam steering, including rapid beam scanning and high beam-pointing accuracy. As SAR missions grow in complexity, the number of channels in PAAs continues to increase in both azimuth and range directions. This expansion necessitates a greater number of T/R modules, resulting in increased antenna mass and power consumption, which in turn places higher demands on the satellite platform’s payload capacity and power supply. Consequently, improving the efficiency of T/R modules and pursuing lightweight antenna designs have become key directions for future development. In contrast, RAs offer excellent beamforming performance, particularly when integrated with digital beamforming (DBF) technology, which provides high-precision beam-pointing capabilities. The combination of RAs and DBF contributes to improved system performance and enhanced imaging quality.

High-resolution, wide-swath imaging places inherently conflicting demands on the antenna [28]. Narrow and stable beams with high sampling favor fine resolution, whereas broad and uniform illumination with strict ambiguity control is needed for large area coverage. In PAA, elevation beam shaping on receive and multi-channel sampling along the flight direction can be realized through digital beamforming and channelized reception, enabling fine resolution while controlling range ambiguities across a wide swath; these benefits, however, increase calibration effort and sensitivity to scan angle. In RA, a large reflective aperture with an electronically controlled feed or a compact feed array can decouple illumination and reception, sweep the footprint, and form narrow receive beams to suppress ambiguities; this generally favors uniform main lobe energy and low side lobes, with agility governed by the feed system and platform steering. Because these behaviors depend on the full three-dimensional radiation pattern—including off-boresight response and steering effects—the present work integrates three-dimensional patterns end-to-end to provide predictions directly relevant to high-resolution, wide-swath design choices between PAA and RA.

In future spaceborne SAR missions, the choice between PAA and RA configurations will depend on factors such as satellite platform complexity, antenna fabrication difficulty, cost, and specific performance requirements. Given the high costs and long development cycles associated with SAR systems, formal validation of operational functionality and ground imaging capabilities through echo simulation is essential prior to deployment. Such validation ensures that the SAR system can accurately detect targets and that the acquired data can be effectively processed, thereby mitigating development risks and reducing overall costs. In SAR echo simulation, accurate incorporation of antenna radiation patterns is critical to ensuring simulation fidelity [29,30]. To achieve echo simulations that closely resemble real-world scenarios, accurate incorporation of antenna characteristics is essential. Antennas with different configurations exhibit distinct radiation behaviors, leading to notable variations in SAR system imaging performance. Traditionally, SAR echo simulations have primarily employed one-dimensional azimuth and range antenna patterns. However, for SAR systems utilizing different antenna architectures, 2D patterns are insufficient to fully capture the spatial characteristics of the antenna beam. Inadequate representation of the 3D radiation pattern may result in discrepancies in simulated imaging outcomes. This paper investigates the imaging differences arising from various SAR antenna configurations. By incorporating full 3D radiation patterns, we simulate the complete SAR imaging process and analyze the resulting imaging characteristics of phased array SAR and reflector SAR systems under varying imaging conditions.

The antenna radiation pattern is a key prior in SAR imaging, simulation, and calibration [31]. While in-flight pattern measurements can refine amplitude modulation, they are costly and impractical for modern systems with hundreds of beam positions; thus, high-fidelity pre-flight echo simulations must incorporate realistic antenna behavior [32]. Conventional simulators often approximate the antenna by separable range/azimuth cuts, which cannot discriminate between architectures: a PAA and an RA with matched 2D cuts yield essentially indistinguishable and often overly optimistic image metrics [29,33]. To address this gap, we integrate full 3D radiation patterns into an end-to-end SAR simulator and use matched-bandwidth PAA and RA exemplars to quantify how genuine 3D effects (off-boresight response, scan-angle dependence, feed-pattern coupling) impact resolution, ambiguity, and side lobe measures under high-resolution, wide-swath conditions. For completeness, we verified that 2D-pattern simulations cannot separate PAA from RA under matched cuts; therefore, our analysis in the main text focuses on 3D-pattern results that are directly relevant to design choices between PAA and RA.

The remainder of this paper is organized as follows: Section 2 provides a brief overview of spaceborne SAR antenna systems, including their fundamental principles and typical design configurations. Section 3 presents the simulation experiments related to the echo design of the spaceborne SAR system and introduces commonly used performance evaluation metrics. Section 4 details the results of the simulation experiments and offers an in-depth analysis of the findings. Finally, Section 5 summarizes the impact of different antenna configurations on the imaging characteristics of spaceborne SAR systems.

## 2. Spaceborne SAR Antenna Systems

Since the launch of the first SAR satellite SEASAT by the United States in 1978, spaceborne SAR has gradually emerged as a focal point in the field of Earth observation. In response, many countries have initiated research programs and developed strategic plans for the deployment of spaceborne SAR satellite systems. Since the early 21st century, several spacefaring nations have successfully launched their own SAR satellites and continuously upgraded their systems [34]. Accompanying this development, SAR antenna technologies have seen significant advancements. Table 1 summarizes the key characteristics and technical specifications of representative spaceborne SAR antennas based on both reflector and phased array configurations.

### 2.1. The Antenna Selection for Spaceborne SAR

The antenna is a key component of spaceborne SAR systems, as its design and performance directly determine imaging quality and resolution. Antenna selection is therefore a comprehensive trade-off among performance, engineering complexity, and mission requirements. PAAs offer high flexibility, enabling agile beam steering, multimode operation, and long mission lifetimes, but at the expense of higher cost and engineering complexity. RAs, by contrast, are mechanically simpler, more mature, and cost-effective, while providing higher radiation efficiency with lower power input.

In practice, platform size strongly influences antenna choice. Most large spaceborne SAR missions (platform mass > 1000 kg) employ PAAs due to their flexible beamforming and rapid pointing capability. In contrast, micro-SAR satellites (platform mass < 1000 kg) often adopt reflector designs, as demonstrated by HJ-1C, TecSAR, SARLupe, and India’s RISAT-2. Specifically, HJ-1C uses a truss-structured multi-beam feed reflector, while TecSAR and RISAT-2 employ phased-feed reflector architectures. Considering cost, weight, and engineering complexity, future micro-SAR satellites may adopt single-beam feed reflectors, multi-beam feed reflectors, or compact active planar phased arrays.

Beyond imaging performance, practical design constraints also play a decisive role in antenna selection. Mass and volume are critical, as PAAs require large numbers of T/R modules that increase payload mass and power demand, whereas deployable RAs achieve lower mass-to-aperture ratios, making them well suited for small satellite platforms. Cost is another key factor: PAAs involve expensive electronics and complex integration, while RAs remain more affordable for resource-limited missions or large constellations. Thermal management and calibration further distinguish the two—distributed modules in PAAs demand sophisticated cooling and continuous amplitude–phase calibration, whereas RAs concentrate heat in fewer amplifiers and mainly rely on feed alignment and platform pointing stability.

Overall, reflector architectures often provide a more balanced trade-off for micro-SAR or cost-sensitive missions, while phased arrays remain indispensable for high-end missions requiring agile beam steering and advanced multimode operation. The main trade-offs between PAAs and RAs are summarized in Table 2.

### 2.2. Reflector Antenna

**Figure 1:**
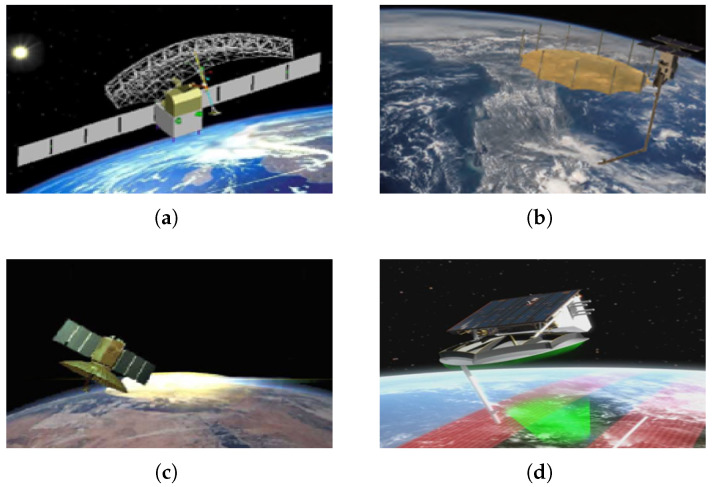
Typical spaceborne SAR systems. (**a**) HJ-1C. (**b**) Capella. (**c**) TecSAR. (**d**) SARLupe.

RAs include several types, such as solid reflectors, parabolic reflectors, truss-based reflectors, and annular reflectors. Due to their inherently limited beam-scanning range, RAs are typically rigidly mounted to the satellite platform. Beam steering is thus achieved by maneuvering the satellite platform itself, allowing for the large rotation angles required for high-resolution imaging. The primary advantage of this configuration is its ability to support continuous scanning, enabling persistent observation over a wide field of view. Moreover, it maintains a consistent peak gain and ensures stable image quality throughout the imaging process. In addition, by leveraging the platform’s ability to tilt laterally, stereo imaging can also be accomplished. Figure 1 illustrates representative spaceborne SAR systems utilizing RA configurations.

RAs achieve wave reflection by redirecting the electromagnetic energy emitted from a feed source off a curved reflective surface. A parabolic reflector, for example, is formed by revolving a parabolic curve symmetrically around its central axis. An RA typically consists of two primary components: the reflector surface and the feed source. Analogous to the principles of optical reflection, RAs convert the feed source’s primary radiation into well-focused secondary radiation. Their radiation patterns are characterized by excellent rotational symmetry, low side lobe levels, and minimal back lobe radiation.

### 2.3. Phased Array Antenna

**Figure 2:**
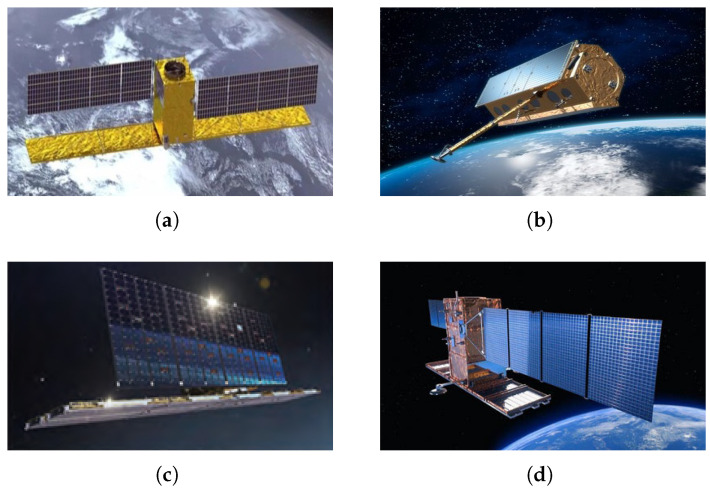
Typical SAR systems employing a phased array configuration. (**a**) Gaofen-3. (**b**) TerraSAR-X. (**c**) IceEye. (**d**) SmartStar SAR payloads.

Compared to RAs, active phased array antennas (PAAs) offer several advantages, including flexible beamforming capabilities, rapid beam steering, spatial filtering and directionality, as well as high reliability. Figure 2 illustrates typical SAR systems employing a phased array configuration. Common radiating elements for SAR PAAs include microstrip antennas [35,36] and waveguide slot antennas. Microstrip antennas are characterized by their low profile, light weight, and ease of integration with electronic systems. However, they typically suffer from lower radiation efficiency and reduced power-handling capability. In contrast, waveguide slot antennas provide higher radiation efficiency than microstrip antennas. Phased array antennas are built from medium-scale line arrays, which serve as the basic units of full-scale aperture arrays. In spaceborne SAR systems, each line array is typically formed by 10 to 20 minimal radiating elements (microstrip patches or waveguide slots), with the excitation of individual elements within the line array being fixed. Each line array then undergoes amplitude–phase control as a unit to achieve high gain and low side lobes in both azimuth and range patterns. By synthesizing multiple line array patterns, the phased array antenna attains higher gain in its cross-section patterns while maintaining lower gain at other angles, which differs markedly from the directional characteristics of reflector antennas.

## 3. Echo Simulation for Spaceborne SAR System

### 3.1. SAR Antenna Simulation

**Figure 3:**
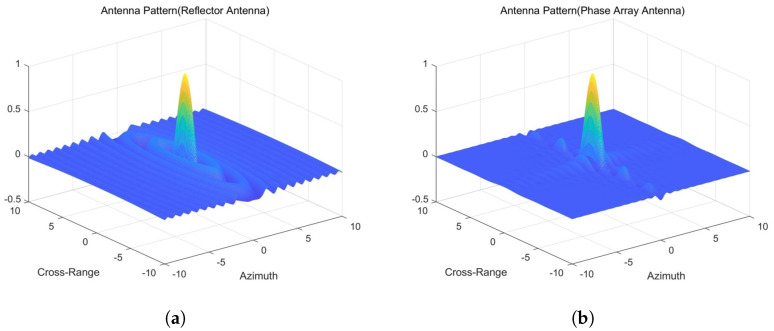
Antenna radiation patterns with matched scales and views. (**a**) Reflector antenna: normalized gain (dB) with contours at −3/−10/−20 dB; annotations mark half-power beamwidth and first side lobe. (**b**) Phased array antenna: same scale and annotations.

We utilize two different antenna patterns for echo simulation. Historically, echo simulation methods have predominantly used 2D directional patterns instead of more accurate 3D patterns, which lead to imprecise results. For phased array antennas, beam synthesis is primarily achieved by multiplying patterns in the azimuth and range directions, whereas reflector antenna systems exhibit circular symmetry. To illustrate the differences between PAA and RA, here, we present their antenna radiation patterns with the same Sinc function in the range and azimuth direction, as shown in Figure 3. Simulating echoes based solely on beam widths in the azimuth and range directions represents a simplified approach. In other directions, the beam pattern lacks symmetry, resulting in discrepancies in echo simulation.

**Figure 4:**
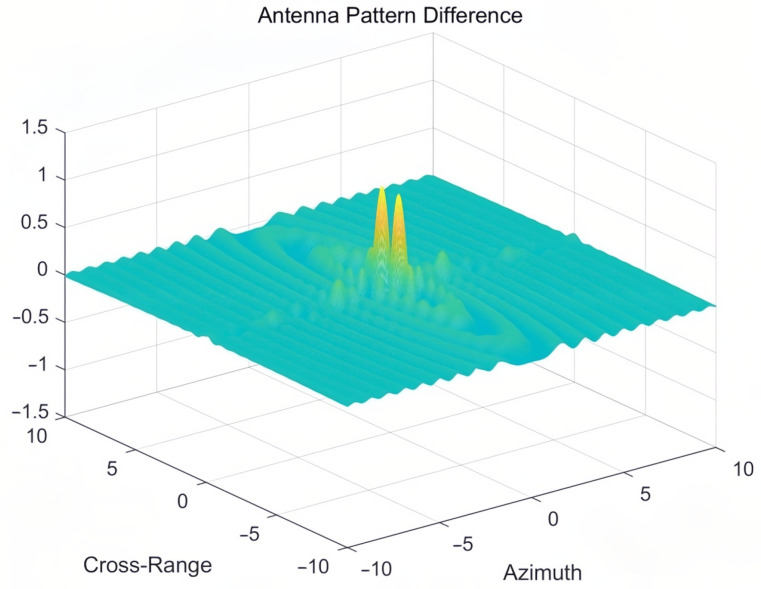
The difference pattern obtained by subtracting the radiation pattern of the RA from that of the PAA.

The differences between the radiation patterns of PAA and RA stem from their distinct structural designs and synthesis principles. In the azimuth direction, PAAs typically exhibit narrower main lobes but higher side lobe levels compared to RA, owing to their electronic beam steering capability and array configuration. Conversely, RAs, characterized by circular symmetry, generally produce wider main lobes with lower side lobe levels. In the elevation direction, PAA may display asymmetrical beam shapes and beam squint effects, which depend on the array design and beamforming algorithms. In contrast, RAs maintain more symmetric elevation patterns, with relatively consistent main lobe shapes and side lobe levels across varying elevation angles. These differences highlight the fundamental contrasts between the two antenna systems in terms of both their physical structures and radiation pattern synthesis methods. Figure 4 displays the differential radiation pattern obtained by subtracting the RA pattern from the PAA pattern, quantitatively characterizing their beamforming distinctions.

The coverage characteristics of PAAs and RAs differ markedly due to their distinct structural and operational principles. PAAs exhibit variability in beamwidth and side lobe levels across both azimuth and elevation angles. Depending on the array configuration and beamforming algorithms, their coverage patterns can be flexibly tailored to specific requirements, enabling adaptive and dynamic beam steering. This electronic steerability allows PAAs to rapidly adjust coverage areas for tracking moving targets or adapting to changing environmental conditions. In contrast, RAs typically provide more uniform and symmetric coverage—especially in the azimuth direction—owing to their circular symmetry. Nonetheless, variations in beamwidth and side lobe characteristics may still arise from differences in reflector geometry and feed design. Generally, RAs offer a symmetric coverage pattern over the entire field of view, which benefits applications demanding consistent wide-area illumination. In summary, PAAs and RAs differ fundamentally in coverage symmetry, adaptability, and beam characteristic variability, reflecting their underlying design philosophies.

### 3.2. Spaceborne SAR System Echo Simulation

The simulation under consideration focuses on stripmap mode in a nadir-view configuration, where the incidence angle is zero. In this scenario, the center of the SAR beam is oriented perpendicularly to the flight direction of the SAR platform.

**Figure 5:**
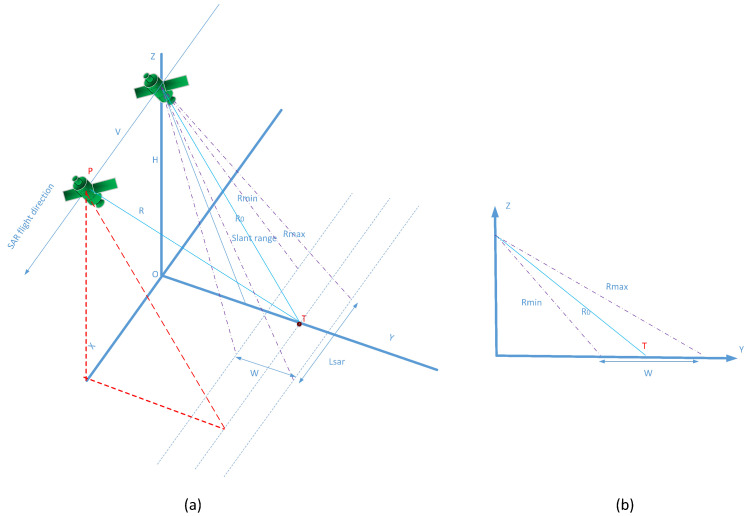
Geometric relationships for radar data acquisition.

We establish a coordinate system XYZ, as illustrated in Figure 5, where the XOY plane represents the ground plane. The SAR platform flies at a constant altitude *H* above the ground and moves uniformly along the *X*-axis with a velocity *V*. Let *P* denote the position vector of the SAR platform, with coordinates (x,y,z), and T denote the position vector of the target, with coordinates $\left(x_{T},y_{T},z_{T}\right)$. Based on the geometric relationship between the target and the SAR platform, the slant range distance *R* can be expressed as

(1) $$R=\left|PT\right|=\sqrt{{\left(x-x_{T}\right)}^{2}+{\left(y-y_{T}\right)}^{2}+{\left(z-z_{T}\right)}^{2}}$$

From Figure 5, y=0, z=0, $z_{T}=0$, make x=vs, where *v* is the platform speed, *s* is the slow time, assuming that $x_{T}=vs$, where *s* represents the SAR platform’s *x*-coordinate for the moment $x_{T}$; and then make $r=\sqrt{H^{2}+y_{T}^{2}}$, which represents the target and the SAR’s vertical obliquity distance, rewrite Equation (1) as

(2) $$\left|PT\right|=R\left(s;r\right)=\sqrt{r^{2}+v^{2}+{\left(s-s_{0}\right)}^{2}}$$

then, R(s;r) denotes the slant distance between the target and the radar at any moment *s*. In general, $v\left|s-s_{0}\right|\ll r$, and so by the Fourier technique expansion, Equation (2) can be written approximately as

(3) $$R\left(s;r\right)=\sqrt{r^{2}+v^{2}\cdot {\left(s-s_{0}\right)}^{2}}\approx r+\frac{v^{2}}{2r}{\left(s-s_{0}\right)}^{2}$$

It can be seen that the slant distance is a function of *s* and *r*. *r* varies from target to target. But when the target is far from the SAR, *r* can be approximated to be constant within the observation band, i.e., $r=R_{0}$. Lsar denotes the synthetic aperture length, and its relationship with the synthetic aperture time Tsar is Lsar=vTsar. Δθ is the radar antenna half-power point beam angle, θ is the beam axis and the *Z* axis angle, that is, the beam angle of view. $R_{\min}$ is the near point distance, $R_{\max}$ is the far point distance, *W* is the width of the mapping strip, and their relationship is

(4) $$\begin{array}{cc} \; & R_{\min}=H\cdot \mathrm{tg}\left(\theta -\frac{\Delta \theta }{2}\right)\\ \; & R_{\max}=H\cdot \mathrm{tg}\left(\theta +\frac{\Delta \theta }{2}\right)\\ \; & W=R_{\max}-R_{\min} \end{array}$$

In SAR operations, signals are transmitted and received in a periodic manner. The transmitter emits chirped pulses at time intervals of $\tau_{l}$, after which the antenna is switched to the receiving mode to capture the corresponding echo signals. When the radar is not in transmission mode, it switches to reception mode to capture the reflected echoes. In airborne applications, the echoes are generally received within the same pulse repetition interval as the transmitted signal. In contrast, for spaceborne radar systems, the large distance between the platform and the target means that echoes from a single pulse may arrive several pulse intervals later—typically between 6 and 10. To simplify the simulation, an spaceborne scenario is assumed in this study.

Assuming $T_{r}$ is the chirp signal duration, the subscript *r* denotes the distance toward. PRF is the repetition frequency, and PRT is the repetition period, equal to 1/PRF. In the receiving sequence, $\tau_{n}=\frac{2R(s;r)}{c}$ denotes the delay of the target echo relative to the transmitting sequence when transmitting the ith pulse. The mathematical expression for the transmit sequence of the radar is the following equation:

(5) $$\begin{array}{cc} \; & s\left(t\right)=\sum_{n=-\infty }^{\infty }p\left(t-n^{*}PRT\right)\\ \; & p\left(t\right)=\mathrm{rect}\left(\frac{t}{T_{r}}\right)e^{j\pi K_{r}t^{2}}e^{j\pi f_{c}t} \end{array}$$

The rect() represents the rectangular signal. $K_{r}$ denotes the range chirp rate and $f_{c}$ is the carrier frequency.

The radar echo signal is jointly influenced by the transmitted waveform, antenna directional pattern, slant range, target radar cross-section (RCS), and surrounding environmental conditions. When environmental effects are ignored, the echo signal from a single point target can be formulated as shown in the following equation:

(6) $$s_{r}\left(t\right)=\sum_{n=-\infty }^{\infty }\sigma wp\left(t-n\cdot PRT-\tau_{n}\right)$$

where σ denotes the radar scattering cross-section of the point target, *w* denotes the bidirectional amplitude weighting of the antenna direction map of the point target, $\tau_{n}$ denotes the delay when the carrier transmits the *n*th pulse and the electromagnetic wave comes back to the carrier again $\tau_{n}=\frac{2R(s;r)}{c}$, and is obtained by banding it into the following equation:

(7) $$\begin{array}{cc} s_{r}\left(t\right) & =\sum_{n=-\infty }^{\infty }\sigma \cdot w\cdot rect\left(\frac{t-n\cdot PRT-\frac{2R(s;r)}{c}}{T_{r}}\right)\cdot \\ \; & \exp[j\pi K_{r}{\left(t-n\cdot PRT-\frac{2R(s;r)}{c}\right)}^{2}]\cdot \\ \; & \exp\left[-j\frac{4\pi }{\lambda }R\left(s;r\right)\right]\cdot \exp\left[j2\pi f_{c}\left(t-n\cdot PRT-\tau_{n}\right)\right] \end{array}$$

Equation (7) represents the single-point target echo signal model. In this equation, $\exp[j\pi K_{r}{\left(t-n\cdot PRT-\frac{2R(s;r)}{c}\right)}^{2}]$ denotes the chirp component, which determines the range resolution, while $\exp[-j\frac{4\pi }{\lambda }R\left(s;r\right)]$ represents the Doppler component, which governs the azimuth resolution. The echo signal for any given pulse can be expressed as shown in the following equation.

(8) $$\begin{array}{cc} s_{r}\left(t,s\right) & =A_{0}w_{r}\left(\tau -2R\left(s;r\right)/c\right)w_{\sigma }\left(s-s_{c}\right)\cdot \exp\left[-j4\pi f_{0}R\left(s;r\right)/c\right]\\ \; & \exp[j\pi K_{r}{\left(\tau -2R\left(s;r\right)/c\right)}^{2}] \end{array}$$

### 3.3. CS Algorithm

Conventional SAR imaging systems typically employ the Range-Doppler algorithm (RDA) [27,37]. However, this approach presents two fundamental limitations. First, while improved Range Cell Migration Correction (RCMC) accuracy can be achieved through longer kernel functions, this comes at the cost of significantly increased computational complexity. Second, the dependency of Second Range Compression (SRC) on the azimuth frequency is difficult to handle effectively, which limits the algorithm’s accuracy when processing SAR data with large squint angles and long apertures. The Chirp Scaling algorithm [38] avoids interpolation operations in RCMC by modulating the chirp signal’s frequency, thereby enabling scaling or shifting of the signal in the range domain.

### 3.4. Evaluation Indicators

#### 3.4.1. Resolution

The ground resolution quantitatively measures the SAR system’s ability to distinguish adjacent targets on the ground and is determined by the half-power main lobe width of the point target impulse response.

Ground resolution in a SAR system consists of both range resolution and azimuth resolution. Specifically, the range resolution is defined as the ground distance corresponding to the half-power width (3 dB) along the range direction, while the azimuth resolution corresponds to the ground distance at the half-power width (3 dB) along the azimuth direction.

#### 3.4.2. Peak Side Lobe Ratio

The peak side lobe ratio (PSLR) is defined as the ratio between the peak intensity of the main lobe of the impulse response function and the peak intensity of the strongest side lobe. This parameter reflects the SAR system’s ability to suppress distortions caused by adjacent point targets. Typically expressed in decibels, the PSLR quantifies the relative strength of the highest side lobe compared to the main lobe in the point target impulse response. The measurement formula for the range peak side lobe ratio is given by

(9) $$PSLR_{r}=20\cdot \log\frac{P_{z}}{P_{mr}}=10\cdot \log\frac{g_{r}^{2}\left(i_{z\;\max}\right)}{g_{r}^{2}\left(i_{\max}\right)}$$

where

(10) $$g_{r}\left(i_{s\;\max}\right)=\max\{\;\begin{array}{c} g_{r}\left(i\right)|1\leq i\leq i_{s\;\min\;l}\cup i_{s\;\min\;r}\leq i\leq N\} \end{array}$$

where $i_{s\;\min\;l}$ is the first minima to the $g_{r}\left(i\right)$ left of the main valve, and $i_{s\;\min\;r}$ is the first minima to the $g_{r}\left(i\right)$ right of the main valve. $g_{r}\left(i_{s\;\max}\right)$ represents the distance to the highest para side lobe peak. The measurement formula for the range PSLR is given by

(11) $$PSLR_{a}=20\cdot \log\frac{P_{za}}{P_{ma}}=10\cdot \log\frac{g_{a}^{2}\left(j_{s\;\max}\right)}{g_{a}^{2}\left(j_{\max}\right)}$$

where

(12) $$g_{a}\left(j_{s\max}\right)=\max\left\{g_{a}\left(j\right)|1\leq j\leq j_{s\;\min\;l}\cup j_{s\;\min\;r}\leq j\leq M\right\}$$

where $g_{a}\left(j_{s\;\max}\right)$ is the highest para side lobe peak in the azimuthal direction. $j_{s\;\min\;l}$ is the first minima on the $g_{a}\left(i\right)$ left side of the main flap. $j_{s\;\min\;r}$ is the first minima on the $g_{a}\left(i\right)$ right side of the main flap. In the radiation pattern, $g_{a}\left(i\right)$ denotes the gain at a specific angle or direction.

#### 3.4.3. Integrated Side Lobe Ratio

The integrated side lobe ratio (ISLR) is defined as the ratio of the energy contained in the main lobe to the total energy in all side lobes. This parameter reflects the SAR system’s ability to suppress distortions caused by nearby distributed targets. Typically expressed in decibels, the ISLR quantifies the relative energy of the side lobes compared to the main lobe in the point target impulse response.

Distance to Integral Bypass Ratio: Calculate the distance to integral $g_{r}\left(k\right)$ bypass ratio by measuring the nearest minima $i_{\max}$ on both sides of the maximum value $i_{\min l}$ and $i_{\min r}$ of the distance interpolated sequence, and by using the following formula:

(13) $$ISLR_{r}=10\cdot \log\frac{\sum_{i=i_{\min\;l}}^{i_{\min\;r}}g_{r}^{2}\left(i\right)}{\sum_{i=i_{\max}-5\left(i_{\max\;r}-i_{\max\;l}\right)}^{i_{\min\;l}-1}g_{r}^{2}\left(i\right)+\sum_{i=i_{\min\;r}+1}^{i_{\max}\;+5\left(i_{\max\;r}-i_{\max\;l}\right)}g_{r}^{2}\left(i\right)}$$

Azimuthal Integral Bypass Ratio: Calculate the azimuthal integral bypass ratio by measuring the position of the nearest minima and on either side of the maximum position $j_{\min l}$ and $j_{\min r}$ of the azimuthally interpolated sequence according to the following formula:

(14) $$ISLR_{\alpha }=10\cdot \log\frac{\sum_{j=j_{\min l}}^{j_{\mathrm{mar}}}g_{\alpha }^{2}\left(j\right)}{\sum_{j=j_{\max}-5\left(j_{\min r}-j_{\min l}\right)}^{j_{\min l}-1}g_{\alpha }^{2}\left(j\right)+\sum_{j=j_{\min r}+1}^{j_{\mathrm{mix}}+5\left(j_{\min}-j_{\min l}\right)}g_{\alpha }^{2}\left(j\right)}$$

#### 3.4.4. Range Ambiguity Signal Ratio

The slant range between a spaceborne SAR system and its targets can extend to several hundred kilometers. Due to the long propagation distance of the radar pulses, the round-trip transmission time often exceeds the pulse repetition period multiple times. Consequently, when the echo signals from the imaging strip arrive at the receiver, additional echoes from other regions—delayed by integer multiples of the pulse repetition period—also reach the receiver via the antenna side lobes. These overlapping signals interfere with the desired echoes, producing what is known as range ambiguity signals. The spatial area where such range ambiguity signals occur is referred to as the range ambiguity region. Consequently, the RASR can be expressed as

(15) $$RASR=\frac{\sum_{m\neq 0}\frac{G_{t}\left(\theta_{am\dot{b},m}\right)\cdot G_{r}\left(\theta_{am\dot{b},m}\right)\cdot \sigma_{0}\left(\theta_{i,m}\right)}{R{\left(\theta_{am\dot{b},m}\right)}^{3}\cdot \sin\theta_{i,m}}}{\frac{G_{t}\left(\theta_{0}\right)\cdot G_{r}\left(\theta_{0}\right)\cdot \sigma_{0}\left(\theta_{i}\right)}{R{\left(\theta_{0}\right)}^{3}\cdot \sin\theta_{i}}}$$

where RASR denotes the range ambiguity-to-signal ratio. Here, $G_{t}$ and $G_{r}$ are the transmit and receive antenna gain patterns, $\sigma_{0}$ is the normalized radar cross-section, and *R* is the slant range distance. The indices *i* and *m* denote the main target and the *m*-th ambiguity region (m≠0), respectively. $\theta_{0}$ and $\theta_{i}$ represent the look and incidence angles of the main beam, while $\theta_{amb,m}$ and $\theta_{i,m}$ correspond to the look and incidence angles of the ambiguity regions. The term sinθ accounts for slant-range projection geometry.

#### 3.4.5. Azimuth Ambiguity Signal Ratio (AASR)

The azimuth ambiguity in the SAR system differs from range ambiguity; it is caused by the pulse sampling mechanism in the azimuth direction. The Doppler spectrum of SAR echo signals along the azimuth is weighted by the antenna’s two-way directional pattern and sampled at the pulse repetition frequency. As a result, Doppler components with frequencies higher than the pulse repetition frequency are aliased back into the central portion of the azimuth spectrum, within the processing bandwidth, causing overlap with the main signal spectrum—an unavoidable phenomenon.

The azimuth ambiguity-to-signal ratio (AASR) is defined as the ratio of the total energy of all azimuth ambiguity signals from imaging targets to the energy of the useful signal. It is expressed as

(16) $$AASR=\frac{\sum_{i\neq 0}\int_{-B_{a}/2}^{B_{a}/2}G_{t}\left(f_{\tau }+i\cdot PRF\right)df_{\tau }}{\int_{-B_{a}/2}^{B_{a}/2}G_{t}\left(f_{\tau }\right)df_{\tau }}$$

In the equation, G(f) represents the effective antenna pattern for transmission and reception; *i* denotes the index of the ambiguity region; $B_{a}$ signifies the processing Doppler bandwidth. In the stripmap mode, the radar platform approximately flies at a constant speed, and the antenna orientation remains unchanged during the imaging process. Hence, the AASR values for all points in the entire imaging area are identical. However, in other SAR operational modes such as scan mode and spotlight mode, the AASR values vary in the azimuth direction.

## 4. Experiments

### 4.1. Simulation Scenario Design

**Figure 6:**
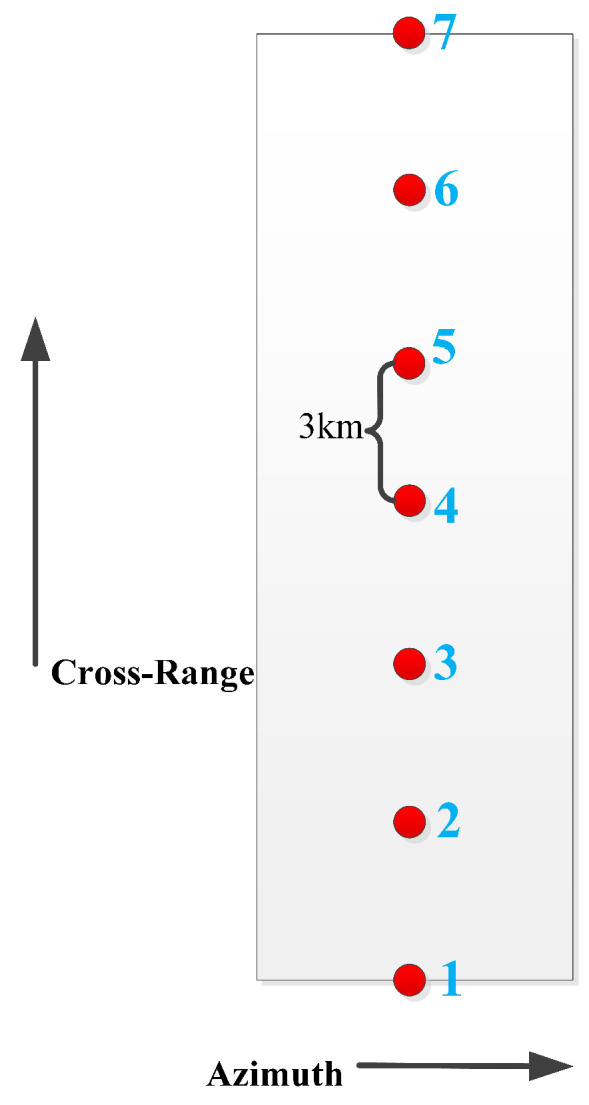
Schematic diagram of point target setting. The red points represent the seven point targets which line up on the same line along the range direction.

**Figure 7:**
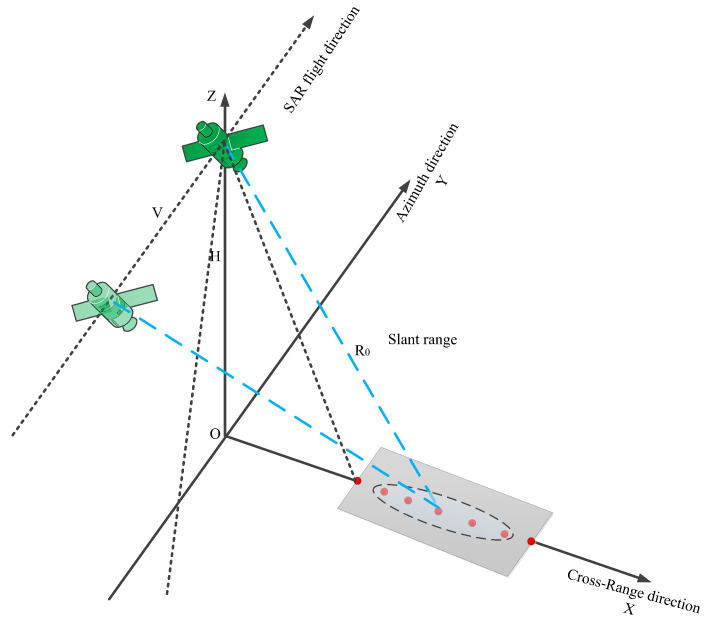
Schematic of SAR system imaging procedure.

We simulate echoes using seven point targets arranged in a grid pattern. Adjacent points in the range direction are spaced 3 km apart, covering a total range width of 18 km. The specific arrangement of the grid is depicted in Figure 6. In the imaging process, the signal intensity received by two points at the same range position is identical. The only difference between different points lies in their imaging time. Figure 7 illustrates the spaceborne SAR imaging chain for the seven calibrated point targets, covering raw data acquisition through to focused image formation.

To expedite simulation speed, we choose to distribute multiple point targets only along the range direction. Sparse grid echo simulation enhances simulation speed, while analyzing the imaging characteristics of point targets facilitates the analysis of imaging effects.

### 4.2. System Simulation Inputs

This section presents simulation experiments based on a representative spaceborne X-band SAR system operating at a center frequency of 9.6 GHz. The specific parameters of the antenna are shown in Table 3. The satellite is assumed to be in a circular orbit at an altitude of 541 km (eccentricity = 0).

Stripmap mode, a typical SAR imaging mode, is employed here for system-level simulation. Due to performance differences between near-range and far-range imaging, SAR system parameters may require distinct configurations. Two representative incidence angles, 25° and 40°, are considered for the simulations. Based on the previously introduced SAR system, the imaging parameters are designed accordingly and detailed in the table below. These parameters are consistently used throughout the subsequent experiments.

### 4.3. Experimental Results and Analysis

#### 4.3.1. Comparison of Imaging Results

**Figure 8:**
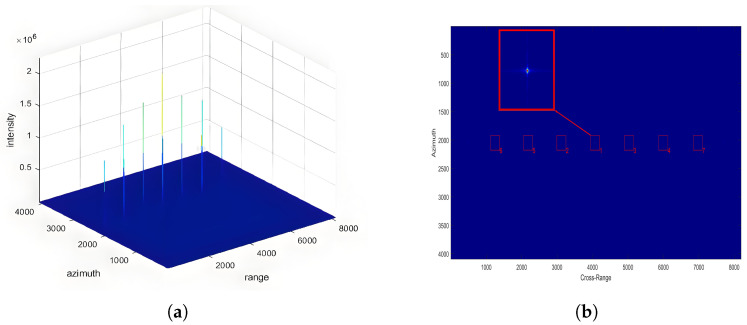
Imaging results of seven point targets at a 25° incidence angle. (**a**) Focused image of the 7-point target array. (**b**) Enlarged view of the 7-point target responses, where brightness ranking indicates the relative intensity of the imaged points.

The parameters described above are used as input to the SAR simulation system for echo signal generation, followed by image reconstruction using the Chirp Scaling (CS) algorithm. The resulting images are presented in Figure 8.

It can be observed that under different incidence angles, all seven point targets are successfully imaged. The point targets are arranged sequentially in the range direction, consistent with the designed input. The numbers in the lower right corner of the small boxes in the figure represent the brightness ranking of the point targets in this scene. Here, brightness refers to the intensity of point target imaging, which is solely related to the weighting of the antenna pattern in the range direction. Under the same incidence angle, the brightness ranking of the imaging results remains consistent across different antenna configurations, which is in line with the design input. The overall difference in the antenna patterns of different antenna configurations is minimal, thus not affecting the overall distribution intensity of the antenna gain in the range direction. At a incidence angle of 25 degrees, it can be observed that the highest brightness point is at the beam center. As the distance from the point target to the beam center increases, the brightness also increases, consistent with theoretical analysis. With the distance from the point target to the beam center in the range direction increasing, the antenna gain decreases, resulting in a decrease in the received intensity as the absolute distance from the beam center increases. Moreover, due to the symmetry of the antenna pattern in the range direction, the brightness ranking remains consistent at equal absolute distances from the beam center, as confirmed by the experimental results.

**Figure 9:**
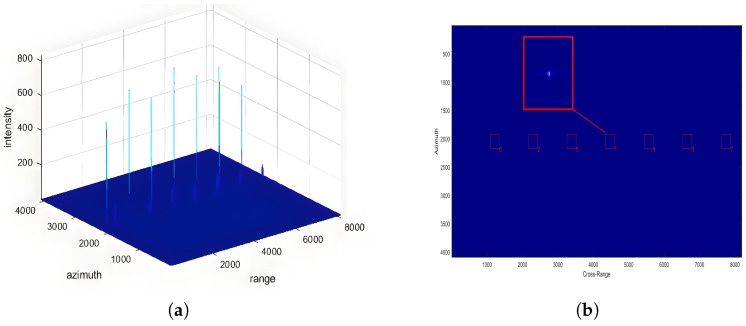
Imaging results of seven point targets at a 45° incidence angle. (**a**) Focused image of the 7-point target array. (**b**) Enlarged view of the 7-point target responses, showing variation in intensity due to antenna side lobe effects at higher incidence angles.

At a 45-degree antenna incidence angle, the brightness of the two points located 6 km from the range beam center is higher than that of the two points at 3 km from the range beam center. The results are shown in Figure 9. This phenomenon occurs because, as the incidence angle increases, the points at 6 km fall within the antenna’s side lobes and thus receive higher antenna gain compared to the points at 3 km, which are closer to the main lobe. From this analysis, it is evident that the experimental results align well with the design parameters and theoretical expectations, thereby validating the accuracy of the simulation.

#### 4.3.2. Comparison of Beam-Center Imaging Results

**Figure 10:**
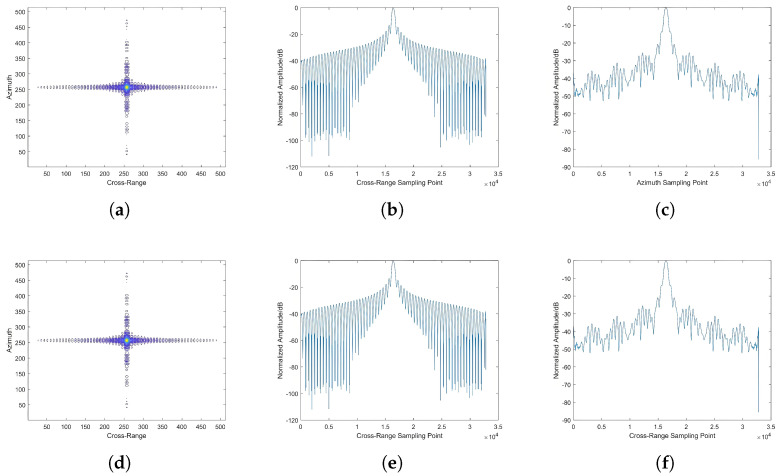
Comparison of point target imaging results at a 25° incidence angle. (**a**) Imaging result of phased array antenna (PAA). (**b**) Range cross-section of PAA. (**c**) Azimuth cross-section of PAA. (**d**) Imaging result of reflector antenna (RA). (**e**) Range cross-section of RA. (**f**) Azimuth cross-section of RA.

**Figure 11:**
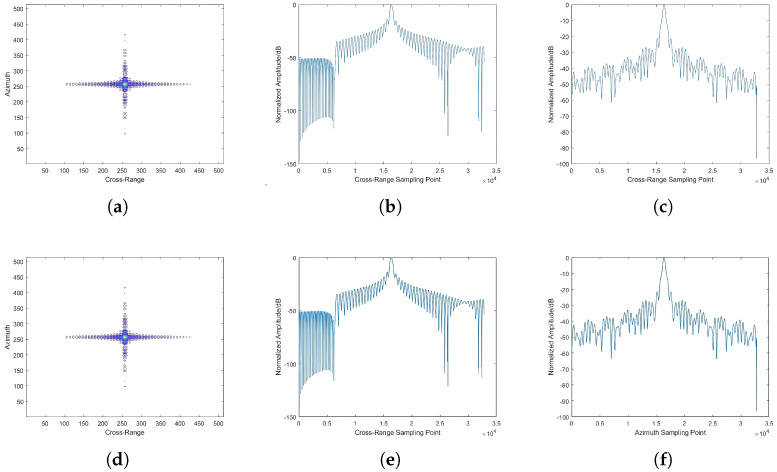
Comparison of point target imaging results at a 45° incidence angle. (**a**) Imaging result of PAA. (**b**) Range cross-section of PAA. (**c**) Azimuth cross-section of PAA. (**d**) Imaging result of RA. (**e**) Range cross-section of RA. (**f**) Azimuth cross-section of RA.

To quantitatively evaluate the beam-center imaging performance across antenna configurations, we isolate the beam-center targets for specialized analysis. The visual comparison (Figure 10 and Figure 11) and quantitative metrics reveal key observations. It is evident that under the same incidence angle, the imaging results of different antenna configurations at the beam center exhibit minimal differences, and the intensity of point targets in the azimuthal and range cross-sections is essentially consistent. For different antenna configurations, the intensity received at the beam center remains constant. The radar signal intensity received at the beam-center point is equivalent to the gain of the antenna azimuthal cross-section pattern. This is also reflected in the imaging results of point targets at the beam center under different incidence angles, where the indicators are essentially consistent, with only minor differences. The differences in this aspect are mainly influenced by the imaging echoes of point targets in other locations. Therefore, it can be concluded that for different SAR configurations, the differences in imaging results at the beam-center position are minimal, but there may be some variations due to the interference from echoes in other areas. Specific indicators are analyzed as shown in Table 4.

#### 4.3.3. Comparison of Resolution

**Figure 12:**
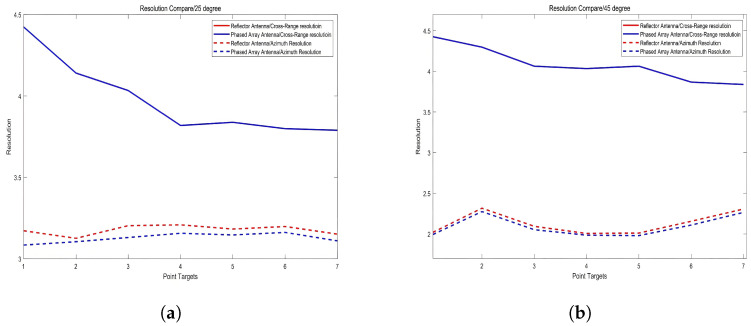
Resolution comparison under different incidence angles. (**a**) Measured range and azimuth resolutions at 25° incidence angle. (**b**) Measured resolutions at 45° incidence angle, highlighting the effect of antenna configuration on azimuth resolution.

This section quantitatively evaluates the resolution characteristics of different antenna architectures across varying incidence angles. Figure 12 presents the measured resolution values for distinct point targets, with the following methodological considerations:

The variation trend of range resolution with respect to different incidence angles remains consistent across various antenna configurations, whereas changes in azimuth resolution are minimal. For both reflector-based and phased-array SAR systems, the azimuth resolution exhibits only slight variations under different incidence angles—within 0.08 m at 25°, and within 0.3 m at 45°. Defined as $\rho_{a}=\frac{\lambda }{2\theta_{B}}$, where $\theta_{B}$ is the beamwidth experienced by ground targets during synthetic aperture formation, azimuth resolution is theoretically independent of the radar beam angle. Minor variations observed are primarily due to differences in antenna pattern weighting across the range and the influence of the CSA imaging process. Notably, the phased-array system achieves slightly better azimuth resolution than the reflector-based system, with differences ranging from 0.02 m to 0.09 m, attributed to its superior energy concentration in both azimuth and range directions, requiring more precise pulse compression alignment in CSA processing. In contrast, range resolution, governed by the radar’s instantaneous bandwidth as $\rho_{r}=\frac{c}{2B}$, is independent of antenna configuration and remains consistent across systems. However, for a given bandwidth, ground range resolution improves with increasing incidence angle—i.e., better resolution at farther ranges—due to geometric projection effects. This trend is observed across different incidence angles, indicating that the antenna pattern has negligible impact on the range resolution, and the processed range profiles are nearly indistinguishable between configurations.

#### 4.3.4. Comparison of Ambiguity

Here, we analyze the ambiguity based on the imaging results obtained at a 25° incidence angle. As an example. The result is summarized in Table 5.

**Figure 13:**
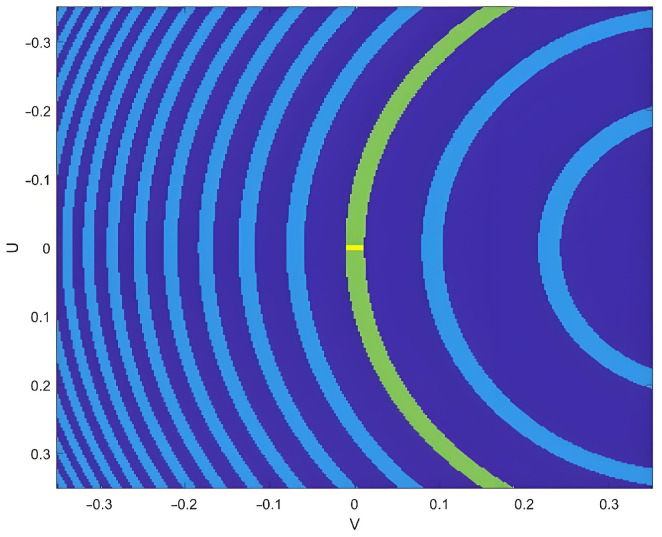
Echo region in the UV coordinate system at a 25° incidence angle. The green region corresponds to the main lobe imaging area, while the yellow strip marks the beam center.

**Figure 14:**
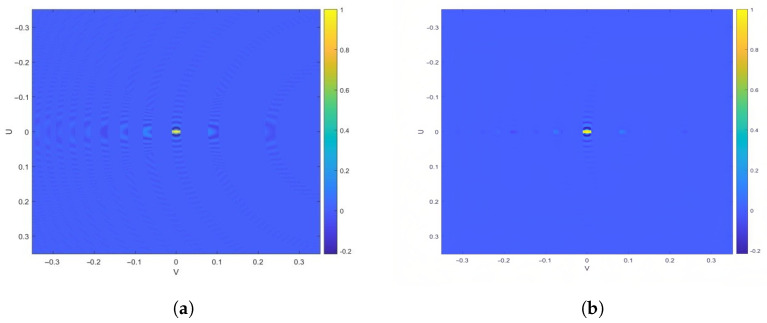
Imaging ambiguity analysis at a 25° incidence angle. (**a**) Echo intensity distribution within the imaging swath. (**b**) Simulated imaging results for the PAA configuration, illustrating side-lobe-related ambiguity effects.

As shown in Figure 13, the locations of ambiguous regions in simulations under different SAR configurations are independent of the antenna type. Furthermore, Figure 14 shows that since the main lobe intensity in the imaging results is significantly stronger than that of the side lobes, the impact of different SAR antenna configurations on RSAR is minimal. According to Section 3.4.4 and Section 3.4.5, RASR is related to the geometric relationship of SAR imaging, while AASR depends on the SAR system’s sampling characteristics and is not affected by the antenna architecture itself. Consequently, the differences between RASR and AASR are small.

The impact of ambiguity on an image is mainly determined by the intensity of the target in the fuzzy zone [39]. Only when they have sufficient scattering intensity will it affect the targets detection within the observation area. From the data in Table 5, we can see that both RASR and AASR are below −20 dB, so their impact on the image can be ignored. The decrease in RASR and AASR mainly relies on optimization the SAR system design itself, such as increasing the strength of the antenna main lobe, optimizing system parameters, and so on. In addition, image processing algorithms can be used to enhance SAR image quality to support target detection or recognition, which requires further research.

#### 4.3.5. Analysis of Other Indicators

**Figure 15:**
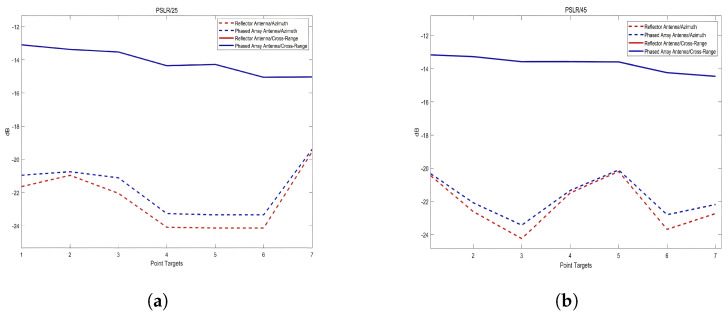
(**a**) Peak side lobe ratio (PSLR); (**b**) integrated side lobe ratio (ISLR).

For different antenna configurations, regardless of the 25° or 45° antenna incidence angles, the imaging results exhibit consistency in the range direction. The differences in PSLR and ISLR are minimal, and these indicators improve with increasing range distance. This indicates that under the same swath, the range direction indicators are independent of the antenna configuration and only correlate with the range distance. The results of the analysis are shown in Figure 15.

**Figure 16:**
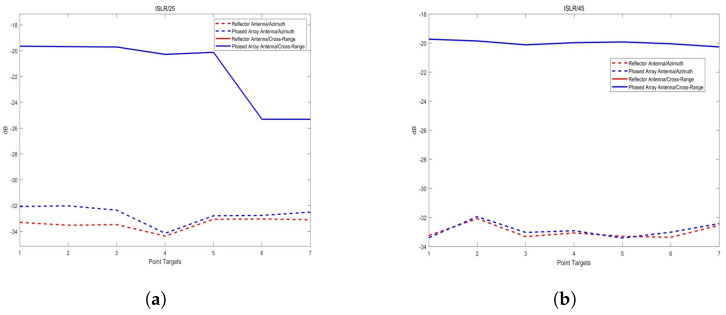
(**a**) Peak side lobe ratio (PSLR); (**b**) integrated side lobe ratio (ISLR).

**Figure 17:**
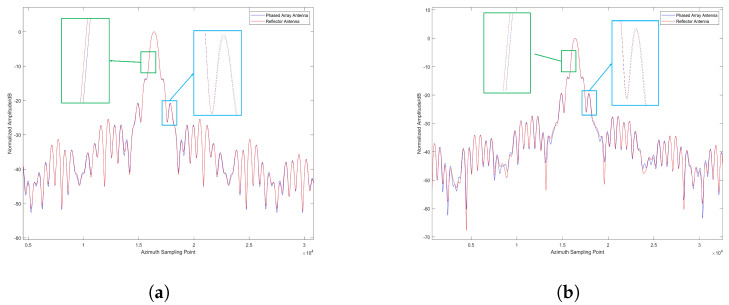
Azimuthal profile comparison of point targets at a 25° viewing angle. (**a**) Profile of Point 1. (**b**) Profile of Point 4. The x-axis is the azimuth sampling point, 250 sampling points equivalent to 1 m.

In terms of azimuthal indicators, whether at 25° or 45° incidence angles, the imaging results of the reflector-based system are superior to those of the phased array system, contrary to the resolution analysis results. The results of the analysis are shown in Figure 16. This is because resolution focuses on the main lobe width in both range and azimuth directions, while PSLR and ISLR reflect the energy difference between the main lobe and side lobes. Although the imaging resolution of the phased array system is better than that of the reflector-based system, the power distribution of the reflector-based system is more uniform at different incidence angles, accumulating more energy in the main lobe. We visualize this difference by extracting the azimuthal profiles of the first and fourth points at a 25° incidence angle. Figure 17 also illustrates that within the main lobe, the PAA curve lies within that of RA for the main lobe, which is consistent with the resolution result (PAA resolution: 3.10 m; RA resolution: 3.12 m), as shown in Table 4. As for the first side lobe peak, the PAA curve is a little higher than that of the RA. The gap between the two curves result in APSLR of RA 0.18 dB higher than APSLR of PAA. After integral calculating, this advantage extends to 0.5 dB in AISLR.

## 5. Conclusions

This paper has systematically investigated the influence of different SAR antenna configurations on the simulation results of a typical spaceborne X-band SAR system by using 3D antenna pattern. Through a series of simulation experiments, key performance indicators such as beam coverage area, radiation pattern characteristics, and ambiguity metrics were analyzed under varying antenna architectures and incidence angles.

The results demonstrate that the SAR antenna configuration significantly affects the final simulated outcomes. Specifically, variations were observed in beamwidth, side lobe levels, coverage symmetry, and ambiguity distribution. These differences arise from the inherent structural and operational distinctions between PAA and RA, impacting parameters such as beam steering flexibility and radiation pattern symmetry.

Importantly, this study reveals that traditional simulation approaches relying solely on azimuth and range direction patterns are insufficiently accurate for capturing the complexities of different SAR antenna systems. Instead, targeted simulation experiments that consider the specific antenna architecture are necessary to obtain realistic and reliable performance assessments, although using the 3D antenna pattern for SAR system simulation will result in additional computational resource. However, the computational efficiency is greatly improved at current, and the increased computational resource can be ignored.

Overall, the findings confirm the necessity and value of the comprehensive simulation framework presented in this work, which accounts for antenna-specific characteristics, thereby providing more meaningful insights for SAR system design and analysis.

## Figures and Tables

**Table 1:** Performance summary of spaceborne SAR antennas.

PAA					
Band	Name	Center Freq. (GHz)	Bandwidth (MHz)	Antenna Type	Antenna Size (m)
P	SeaSat	1.275	19	Microstrip patch	10.7 × 2.2
	SIR-A	1.275	6	Microstrip patch	9.4 × 2.16
	JERS-1	1.275	15	Microstrip patch	11.9 × 2.2
	PALSAR	1.270	28	Microstrip patch	–
	NovaSAR-S	3.2	200	Microstrip patch	3 × 1
C	ERS-1/2	5.3	15.55	Waveguide slot array	11 × 1.3
	RADARSAT-1	5.3	30	Microstrip patch	15 × 1.5
	RADARSAT-2	5.405	100	Microstrip patch	15 × 1.37
	ENVISAT	5.331	124	Microstrip patch	10 × 1.3
	SENTINEL-1	5.405	100	Waveguide slot array	12.3 × 0.9
	GaoFen-3	5.4	240	Waveguide slot array	15 × 1.5
X	Cosmo-SkyMed	9.6	400	Microstrip patch	5.7 × 1.4
	TerraSAR-X	9.65	300	Waveguide slot array	4.8 × 0.7
	Paz	9.65	300	Waveguide slot array	4.8 × 0.7
	ICEye	9.65	300	–	3.25 × 0.4
	StriX	9.65	300	Waveguide slot array	4.9 × 0.7
RA					
S	HJ-1C	3.2	80	Reticulated parabolic antenna	6 × 2.8 (spread caliber)
X	TecSAR	9.59	200	Umbrella reflecting surface	3 m diameter
	SARLupe	9.65	–	Fixed parabolic antenna	3.3 × 2.7
	RISAT	9.59	–	Umbrella reflecting surface	3.6 m diameter
	Capella	9.65	500	Reticulated reflective surface	3.5 m diameter

**Table 2:** Key trade-offs between phased array antennas (PAAs) and reflector antennas (RAs).

Aspect	Phased Array Antennas (PAAs)	Reflector Antennas (RAs)
Beam steering	Agile electronic steering	Limited, platform-dependent
Resolution	Slightly higher azimuth resolution	More uniform beam, lower side lobes
Mass and power	Heavy, high power demand	Lower mass-to-aperture ratio
Cost	Expensive, complex integration	Cheaper, mechanically simpler
Calibration	Continuous multi-channel calibration	Mainly feed alignment

**Table 3:** Parameters.

Parameter	Value
left frequency	9600 MHz
Antenna beamwidth	0.39° (Azimuth) × 1.65° (Range)
Antenna efficiency	56.8%
Antenna maximum gain	44.4 dB

**Table 4:** Abbreviated imaging performance metrics under different SAR architectures and incidence angles. Note: VA = Incidence Angle; SAR Type = SAR System Architecture; RR = Range Resolution; AR = Azimuth Resolution; APSLR = Azimuth Peak Side Lobe Ratio; RPSLR = Range Peak Side Lobe Ratio; AISLR = Azimuth Integrated Side Lobe Ratio; RISLR = Range Integrated Side Lobe Ratio; 2D-ISLR = 2D Integrated Side Lobe Ratio.

VA	SAR Type	RR	AR	APSLR	RPSLR	AISLR	RISLR	2D-ISLR
25 degrees	Phased Array	4.14	3.10	−13.64	−13.36	−24.02	−19.68	−14.27
	Reflector	4.14	3.12	−13.82	−13.36	−24.52	−19.68	−14.87
	Difference	0	−0.02	0.18	1.64	0.50	−8.93	0.60
45 degrees	Phased Array	4.03	1.98	−21.33	−13.58	−32.90	−19.97	−17.77
	Reflector	4.03	2.00	−21.48	−13.58	−33.05	−19.97	−17.93
	Difference	0	−0.02	0.15	0	0.15	0	0.15

**Table 5:** Comparison of RASR and AASR under different SAR antenna architectures at 25° incidence angle.

		Phased Array (dB)	Reflector (dB)
25°	RASR	−32.0	−32.0
	AASR	−24.6	−24.6

## Data Availability

The data used in this paper are all simulation data, and the input for data simulation is detailed in Section 4.2.
